# Evaluation of the Feasibility, Safety, and Effectiveness of a Tailored Enhanced Recovery After Surgery Protocol for Gastroepiploic Vascularized Lymph Node Transfer in Breast Cancer‐Related Upper Extremity Lymphedema: A Preliminary Retrospective Comparative Study

**DOI:** 10.1002/micr.70294

**Published:** 2026-09-20

**Authors:** Federico Lo Torto, Bilel Dribine, Alessia Pagnotta, Andrea Loreti, Giuseppe Cavallaro

**Affiliations:** ^1^ Unit of Plastic and Reconstructive Surgery, Department of Medical, Oral, and Biotechnological Sciences University “G. d'Annunzio” of Chieti–Pescara Chieti Italy; ^2^ Unit of Plastic and Reconstructive Surgery, Department of Surgery, Policlinico Umberto I Sapienza University of Rome Roma Italy; ^3^ Department of Plastic and Reconstructive Surgery Azienda Ospedaliera San Giovanni‐Addolorata Rome Italy; ^4^ Unit of General Surgery, ICOT Hospital (LT), Department of Surgery “Sapienza” University of Rome Roma Italy

**Keywords:** breast cancer‐related lymphedema, enhanced recovery, ERAS, gastroepiploic flap, lymphedema, perioperative care, vascularized lymph node transfer

## Abstract

**Background:**

Enhanced recovery after surgery (ERAS) protocols are multimodal perioperative pathways designed to reduce surgical stress, accelerate recovery, and improve resource utilization. Although ERAS principles have been increasingly adopted in microsurgery, their application in vascularized lymph node transfer (VLNT), particularly using gastroepiploic flaps, remains poorly defined. The aim of this study was to evaluate the feasibility, safety, and effectiveness of a tailored ERAS protocol in patients undergoing gastroepiploic VLNT for breast cancer‐related upper extremity lymphedema.

**Patients and Methods:**

A retrospective comparative study was conducted in patients with unilateral breast cancer‐related upper extremity lymphedema (ISL Stage II–III) undergoing gastroepiploic vascularized lymph node transfer after failure of at least 6 months of conservative treatment. Patients were managed either with a conventional perioperative protocol, including nasogastric tube placement, delayed oral intake, delayed mobilization, opioid‐based analgesia, and delayed initiation of complete decongestive therapy (CDT), or with a tailored ERAS‐VLNT protocol incorporating early oral intake, early mobilization, opioid‐sparing analgesia, avoidance of nasogastric decompression, and CDT initiation from postoperative Day 7. Outcomes included circumferential reduction rate, LYMQOL score, infection rate, flap‐related complications, length of hospital stay, and hospitalization costs.

**Results:**

A total of 45 patients were included, with 29 in the ERAS group and 16 in the conventional group. Baseline demographic and clinical characteristics were comparable between groups. The ERAS group demonstrated a significantly shorter hospital stay (2.34 ± 0.48 vs. 5.56 ± 0.51 days, *p* < 0.001) and lower estimated hospitalization costs (€1400 vs. €3300, *p* < 0.001). Flap survival was 100% in both groups, with no flap‐related complications. Circumferential reduction rate was comparable between groups (44.39% ± 10.20% vs. 47.53% ± 8.26%, *p* = 0.278), as were LYMQOL scores at 12 months (8.21 ± 0.73 vs. 7.93 ± 0.70, *p* = 0.236) and infection rates (2.62 ± 0.90 vs. 2.73 ± 0.96 episodes/year, *p* = 0.71).

**Conclusion:**

An ERAS‐based protocol tailored to VLNT is feasible and safe, and is associated with improved perioperative recovery and reduced healthcare costs without evidence of compromised surgical or functional outcomes. These findings support the integration of ERAS principles into lymphatic microsurgery and warrant further prospective validation.

## 1 Introduction

Breast cancer–related lymphedema (BCRL) is a chronic and often progressive condition that significantly impairs functional capacity and quality of life, affecting up to 20%–30% of patients following breast cancer treatment (DiSipio et al. 2013; Rockson 2018).

Clinically, BCRL is characterized by limb swelling, recurrent infections, discomfort, and progressive tissue changes, resulting in substantial long‐term morbidity and increased healthcare burden. Furthermore, variability in the quality of available information on lymphedema management has been reported, highlighting the need for standardized and evidence‐based approaches (Lo Torto et al. 2021).

In recent years, physiologic microsurgical approaches such as vascularized lymph node transfer (VLNT) have emerged as effective treatment strategies, particularly in advanced‐stage disease where lymphatic channels are severely compromised (Cheng et al. 2013; Schaverien and Coroneos 2019; Ozturk et al. 2016; Becker et al. 2006). Among the various techniques described, gastroepiploic vascularized lymph node transfer with orthotopic axillary inset has gained increasing attention due to its ability to restore physiological lymphatic drainage while simultaneously addressing axillary scarring and improving venous outflow (Manrique et al. 2020; Ciudad et al. 2017).

Despite advances in surgical technique, perioperative management in lymphatic microsurgery remains heterogeneous and largely based on institutional experience. In our institution, conventional perioperative management following gastroepiploic vascularized lymph node transfer traditionally included routine nasogastric tube placement, delayed oral intake, cautious mobilization, and delayed initiation of rehabilitation protocols. These measures reflected a conservative postoperative approach aimed at minimizing the risk of flap compromise and donor‐site complications.

Enhanced Recovery After Surgery (ERAS) programs represent a paradigm shift in perioperative care, based on multimodal, evidence‐based strategies aimed at reducing surgical stress and accelerating postoperative recovery. Initially developed in colorectal surgery, ERAS pathways have progressively been adopted across multiple surgical specialties, including breast reconstruction and free flap microsurgery, where they have demonstrated reductions in hospital length of stay, complications, and healthcare costs (Ljungqvist et al. 2017; Gustafsson et al. 2019; Greco et al. 2014; Temple‐Oberle et al. 2017; Brindle et al. 2020).

However, existing ERAS protocols in microsurgery have been primarily designed for reconstructive procedures focused on tissue replacement rather than restoration of lymphatic function. As such, they do not incorporate lymphatic‐specific considerations, such as the integration of postoperative rehabilitation with complete decongestive therapy (CDT) or the optimization of lymphatic and venous outflow following orthotopic inset.

To date, no ERAS protocol has been specifically tailored to lymphatic microsurgery, and its application to vascularized lymph node transfer has not been systematically investigated.

We hypothesized that a tailored ERAS protocol adapted to VLNT (ERAS‐VLNT) could allow a less invasive perioperative course—characterized by avoidance of nasogastric decompression, early oral intake, early mobilization, opioid‐sparing analgesia, and earlier initiation of CDT—without compromising flap viability or functional outcomes.

The aim of this study was to evaluate the feasibility, safety, and effectiveness of a novel ERAS‐based perioperative protocol specifically designed for vascularized lymph node transfer, and to compare it with conventional perioperative management.

## 2 Patients and Methods

This retrospective, single‐center observational study included consecutive patients diagnosed with breast cancer–related lymphedema (BCRL) and treated at our institution between August 2019 and January 2025. Patients were allocated to ERAS or conventional management according to the perioperative protocol in use at the time of surgery. The ERAS‐VLNT protocol was progressively introduced in January 2023 and subsequently became the standard perioperative pathway at our institution. Therefore, patients treated before January 2023 were included in the conventional group, whereas those treated thereafter were managed according to the ERAS‐VLNT protocol.

Diagnosis was established through clinical evaluation and lymphoscintigraphy. Lymphedema severity was classified according to the International Society of Lymphology (ISL) staging system. In selected cases, particularly when clinical or lymphoscintigraphic findings were inconclusive, magnetic resonance lymphangiography (MRL) was performed to further evaluate lymphatic anatomy and function.

Patients were eligible for inclusion if they presented with unilateral secondary upper limb lymphedema following breast cancer treatment, including axillary lymph node dissection or sentinel lymph node biopsy. Additional inclusion criteria comprised ISL Stage II or III lymphedema, abnormal lymphoscintigraphic findings, a minimum of 6 months of unsuccessful conservative management, and a minimum follow‐up of 12 months.

All included patients underwent vascularized lymph node transfer and were retrospectively divided into two groups based on perioperative management: those treated according to an Enhanced Recovery After Surgery (ERAS) protocol and those managed with a conventional perioperative approach.

Exclusion criteria included bilateral lymphedema, local tumor recurrence or distant metastases, acute limb infection, and deep venous thrombosis.

### 2.1 Surgical Technique

Informed consent was obtained from all patients prior to surgery. All procedures were performed as delayed lymphatic reconstruction, and no simultaneous breast reconstruction or additional major procedures were carried out.

All patients underwent vascularized lymph node transfer (VLNT) using a gastroepiploic lymph node flap, harvested laparoscopically and transferred to the axillary region with orthotopic inset (Lo Torto et al. 2024). Flap harvest was performed through a minimally invasive laparoscopic approach, in accordance with principles aimed at reducing donor‐site morbidity (Elia et al. 2022; Ciudad, Manrique, Bustos, Perez Coca, et al. 2020).

The right gastroepiploic vascular arcade was identified along the greater curvature of the stomach, and a lymph node–bearing segment of the omentum was carefully dissected. Particular attention was paid to preserving the integrity of the vascular pedicle and minimizing thermal injury to surrounding lymphatic structures. The flap included the gastroepiploic artery and vein together with associated lymphatic tissue, with an average pedicle length ranging between 5 and 8 cm.

A two‐team approach was systematically adopted, allowing simultaneous donor‐site harvest and recipient‐site preparation. The recipient site was accessed through the previous axillary scar, where extensive adhesiolysis and scar release were performed. This step was considered essential to decompress the neurovascular structures and to improve both venous and lymphatic outflow, in line with previously described orthotopic VLNT technique (Lo Torto et al. 2024; Ciudad, Manrique, Bustos, Perez Coca, et al. 2020).

Recipient vessels were identified among branches of the thoracodorsal or lateral thoracic system. Microsurgical end‐to‐end anastomoses were performed under magnification. The flap was inset in an orthotopic configuration within the axilla, without tension or compression, with the aim of restoring physiological lymphatic drainage pathways. A closed‐suction drain was routinely placed to prevent seroma formation and avoid compression of the vascular pedicle, thereby ensuring optimal flap perfusion, in accordance with microsurgical safety principles within an ERAS framework.

This approach allows not only lymph node transfer but also functional restoration of the axillary region through scar release and improvement of local hemodynamics, representing a potential advantage of orthotopic VLNT compared with heterotopic techniques.

A detailed demonstration of flap harvest, microvascular anastomosis, orthotopic inset, and intraoperative perfusion assessment is provided in the Supporting Information Video S1.

Flap monitoring was performed through clinical assessment and handheld Doppler examination every 2 h in the early postoperative period and at regular intervals thereafter. Ultrasonography was not routinely used for postoperative flap monitoring. Instead, the vascular pedicle was carefully identified intraoperatively, and its anatomical course was marked on the skin using a sterile marker to allow reliable postoperative localization with a handheld Doppler device. This approach enabled consistent assessment of flap perfusion despite the buried position of the lymph node flap throughout the postoperative monitoring period.

Flap survival was defined as the absence of partial or total necrosis, no requirement for surgical re‐exploration, and preserved flap viability throughout follow‐up, with long‐term flap viability routinely confirmed by lymphoscintigraphy performed at the 12‐month follow‐up.

### 2.2 ERAS‐VLNT Protocol

**FIGURE 1:**
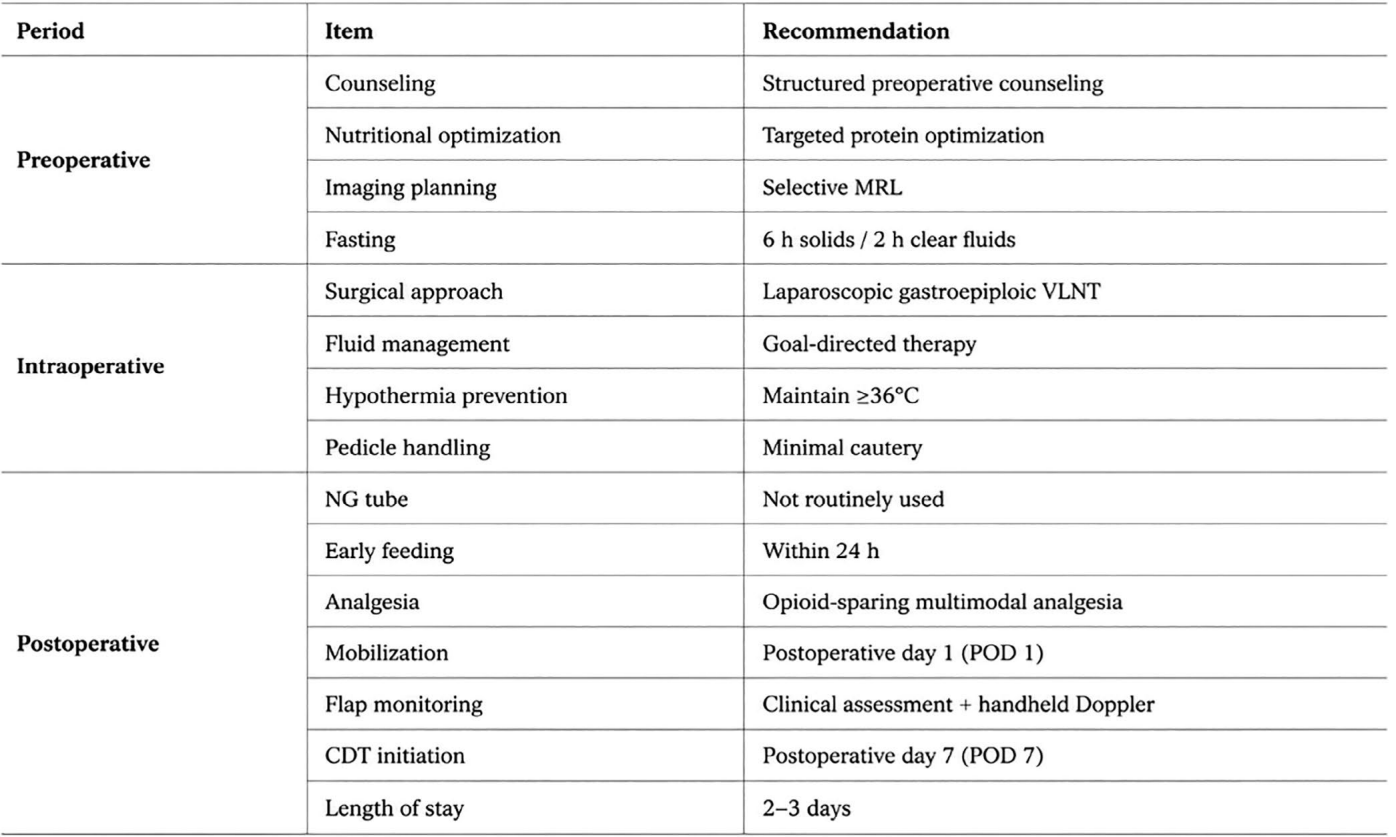
ERAS‐VLNT protocol. Schematic representation of the Enhanced Recovery After Surgery (ERAS) protocol specifically adapted for vascularized lymph node transfer (VLNT). The protocol is structured across the preoperative, intraoperative, and postoperative phases. Preoperative measures include structured patient counseling, nutritional optimization, selective imaging with magnetic resonance lymphangiography (MRL), and shortened fasting (6 h for solids and 2 h for clear fluids). Intraoperative management is based on a minimally invasive laparoscopic approach for gastroepiploic lymph node harvest, goal‐directed fluid therapy, maintenance of normothermia (≥ 36°C), and atraumatic pedicle handling with minimal cautery. Postoperative care includes avoidance of routine nasogastric tube placement, early oral intake within 24 h, opioid‐sparing multimodal analgesia, early mobilization from postoperative Day 1 (POD1), handheld Doppler flap monitoring, and initiation of complete decongestive therapy (CDT) from postoperative Day 7 (POD7). These measures are associated with a marked reduction in hospital length of stay to approximately 2–3 days. This protocol represents the first ERAS pathway specifically designed for lymphatic microsurgery, integrating enhanced recovery principles with microsurgical safety.

Perioperative management in the ERAS group was standardized according to a novel protocol specifically designed for lymphatic microsurgery (ERAS‐VLNT), which represents the central innovation of this study (Figure 1).

The protocol was developed based on established ERAS principles, including preoperative patient education, minimization of fasting, and optimization of nutritional status, and adapted to the specific requirements of microsurgical lymph node transfer.

Preoperatively, patients underwent structured counseling regarding the surgical procedure, postoperative expectations, and early mobilization strategies. Prolonged fasting was avoided, and carbohydrate loading was encouraged when appropriate.

Intraoperative management included goal‐directed fluid therapy to maintain adequate perfusion while avoiding fluid overload, which may negatively affect flap microcirculation, along with maintenance of normothermia and atraumatic tissue handling.

Intraoperative and postoperative fluid therapy followed a goal‐directed, restrictive but nonhypovolemic approach aimed at maintaining normovolemia and preserving flap perfusion rather than focusing solely on systemic blood pressure. Balanced crystalloid solutions were used, and no colloids were administered. Intravenous fluids were administered during the first 24 h, after which early oral intake was initiated, allowing prompt discontinuation of intravenous fluid therapy. This strategy aimed to avoid both fluid overload and hypovolemia, which may adversely affect microvascular perfusion.

Postoperatively, the ERAS‐VLNT protocol introduced several key modifications compared with conventional management. Routine use of nasogastric tubes was avoided. Oral intake was resumed within the first 24 h in all patients, reflecting a shift from traditional conservative approaches that delay feeding due to concerns regarding gastrointestinal recovery.

Early mobilization was implemented from postoperative Day 1 in a controlled and progressive manner, allowing patients to ambulate independently. Early mobilization was introduced to enhance recovery while maintaining flap safety.

Postoperative analgesia was administered on an as‐needed basis. Intravenous paracetamol (1000 mg up to three times daily) was used as first‐line therapy, with nonsteroidal anti‐inflammatory drugs administered as needed. No routine use of opioids or patient‐controlled analgesia was adopted.

Flap monitoring was performed through clinical assessment and handheld Doppler examination, without the need for prolonged intensive care unit monitoring.

Complete decongestive therapy (CDT) was initiated from postoperative Day 7, allowing early functional rehabilitation without compromising flap viability. This timing was selected because compression therapy was applied to the affected limb while avoiding direct compression of the axillary region containing the transferred lymph node flap and microvascular anastomoses. Therefore, early CDT could be resumed while minimizing the risk of mechanical stress on the flap pedicle and vascular connections.

Perioperative thromboprophylaxis consisted of mechanical and pharmacological measures. Anti‐embolism stockings were routinely applied intraoperatively. Low‐molecular‐weight heparin (enoxaparin) was initiated on the first postoperative day at a weight‐adjusted prophylactic dose and continued for 28 days. Early mobilization from postoperative Day 1 represented an additional component of the thromboprophylaxis strategy.

### 2.3 Conventional Perioperative Protocol

In the conventional perioperative protocol, a more conservative approach was adopted.

A nasogastric tube was routinely placed intraoperatively and maintained until gastric output decreased to less than 40 mL. Oral intake was delayed until the return of bowel function, typically around 48 h postoperatively.

Mobilization was postponed, with patients generally remaining in bed for at least 48 h.

Fluid administration ranged from 30 to 50 mL/kg per day and was typically continued beyond the first 24 h postoperatively, as oral intake was delayed until approximately 48 h after surgery. Fluid management was based on standard perioperative practice, without the use of goal‐directed strategies, and was not specifically tailored to flap perfusion parameters.

Analgesia was managed using continuous elastomeric infusion devices delivering morphine.

Complete decongestive therapy (CDT) was initiated no earlier than 15 days postoperatively, typically from postoperative Day 16, due to concerns regarding potential compromise of the axillary flap.

### 2.4 Outcome Measures

Primary outcomes included co‐primary endpoints: circumferential reduction rate (CRR), patient‐reported quality of life assessed using the LYMQOL questionnaire, and soft tissue infection rates.

Circumferential measurements were obtained for both the affected limb (AL) and the contralateral healthy limb (HL) preoperatively and monthly after surgery at five standardized levels: 10 cm above the elbow, at the elbow, 10 cm below the elbow, at the wrist, and at the midhand.

CRR was defined as the percentage reduction in limb circumference difference between the affected and healthy limb and was calculated using the following formula:

$$\mathrm{CRR}\;\left(\%\right)=\left[1-\left(\text{post}-\mathrm{op}\;\mathrm{AL}-\mathrm{HL}\right)/\left(\mathrm{pre}-\mathrm{op}\;\mathrm{AL}-\mathrm{HL}\right)\right]\times 100.$$

Soft tissue infection incidence was defined as the number of infectious episodes per year requiring antibiotic therapy and diagnosed based on clinical signs, including erythema, swelling, local warmth, and pain of the affected limb. Infection rates were assessed both before and after treatment, with postoperative evaluation at 12 months following VLNT.

LYMQOL questionnaire scores were collected 1 month prior to surgery and at 12 months following VLNT. The LYMQOL instrument comprises four domains—symptoms, body image, function, and psychological well‐being—for a total of 27 items. Each item is scored on a scale from 1 to 4, with the overall score ranging from 0 to 10, where higher values indicate better quality of life.

Secondary outcomes included flap survival, flap‐related complications (including arterial or venous thrombosis, venous congestion, partial ischemia, and need for surgical re‐exploration), donor‐site complications, length of hospital stay, nasogastric tube use, timing of oral intake, timing of mobilization, timing of complete decongestive therapy (CDT) initiation, analgesic requirements, and estimated hospitalization costs.

Estimated hospitalization costs were calculated based on the average daily cost of surgical inpatient care derived from national healthcare data (including reports from the Italian Ministry of Health and AGENAS) and adjusted according to the length of hospital stay for each patient.

Postoperative pain was not systematically quantified using standardized scales but was indirectly assessed through analgesic requirements.

All postoperative outcomes were assessed at 12 months following VLNT, which represented the minimum follow‐up required for inclusion in the study.

### 2.5 Statistical Analysis

Continuous variables were expressed as mean ± standard deviation and compared between groups using Welch's *t*‐test. Paired comparisons between preoperative and postoperative values were performed using the paired *t*‐test. Categorical variables were analyzed using Fisher's exact test due to the small sample size and low expected frequencies. A *p*‐value < 0.05 was considered statistically significant.

## 3 Results

### 3.1 Baseline Characteristics

From August 2019 to January 2025, a total of 48 patients diagnosed with breast cancer–related lymphedema (BCRL) were treated at our center. Of these, 45 patients met the inclusion criteria and were retrospectively analyzed, with a minimum follow‐up of 12 months.

The cohort included 29 patients in the ERAS group and 16 in the conventional care group. Baseline demographic and clinical characteristics are summarized in Table 1.

**TABLE 1:** Baseline patient characteristics.

Variable	ERAS protocol (*n* = 29)	Control (*n* = 16)	*p*
Number of patients	29	16	—
Age (years)	54 (41–66)	56 (47–64)	0.42
BMI (kg/m^2^)	32.1 (24–38)	31.7 (22–37)	0.71
Hypertension, *n* (%)	6 (20.7%)	2 (12.5%)	0.68
Diabetes, *n* (%)	1 (3.4%)	2 (12.5%)	0.27
Smoking (> 10 cigarettes/day), *n* (%)	5 (17.2%)	6 (37.5%)	0.12
Breast‐conserving surgery + SLNB, *n* (%)	6 (20.7%)	2 (12.5%)	0.68
Mastectomy + ALND, *n* (%)	23 (79.3%)	14 (87.5%)	0.68
Radiotherapy, *n* (%)	29 (100%)	16 (100%)	—
ISL Stage II, *n* (%)	5 (17.2%)	2 (12.5%)	1.00
ISL Stage III, *n* (%)	24 (82.8%)	14 (87.5%)	1.00

*Note:* Values are presented as mean (range) or number (percentage). Continuous variables were compared using Welch's *t*‐test, while categorical variables were analyzed using Fisher's exact test. A *p*‐value < 0.05 was considered statistically significant.

Abbreviations: ALND, axillary lymph node dissection; BMI, body mass index; ISL, International Society of Lymphology; SLNB, sentinel lymph node biopsy.

No statistically significant differences were observed between the two groups in terms of age, body mass index (BMI), comorbidities, or lymphedema stage, indicating that the cohorts were comparable at baseline.

### 3.2 Intraoperative Outcomes

All procedures were successfully completed without intraoperative complications or technical difficulties. Intraoperative characteristics were comparable between the ERAS and conventional groups.

### 3.3 Surgical and Functional Outcomes

Flap survival was 100% in both groups. No flap‐related complications, including arterial or venous thrombosis, venous congestion, partial ischemia, or need for surgical re‐exploration, were observed. No donor‐site complications occurred.

At 12 months of follow‐up, both groups demonstrated significant clinical improvement compared with baseline (*p* < 0.001 for all comparisons).

**FIGURE 2:**
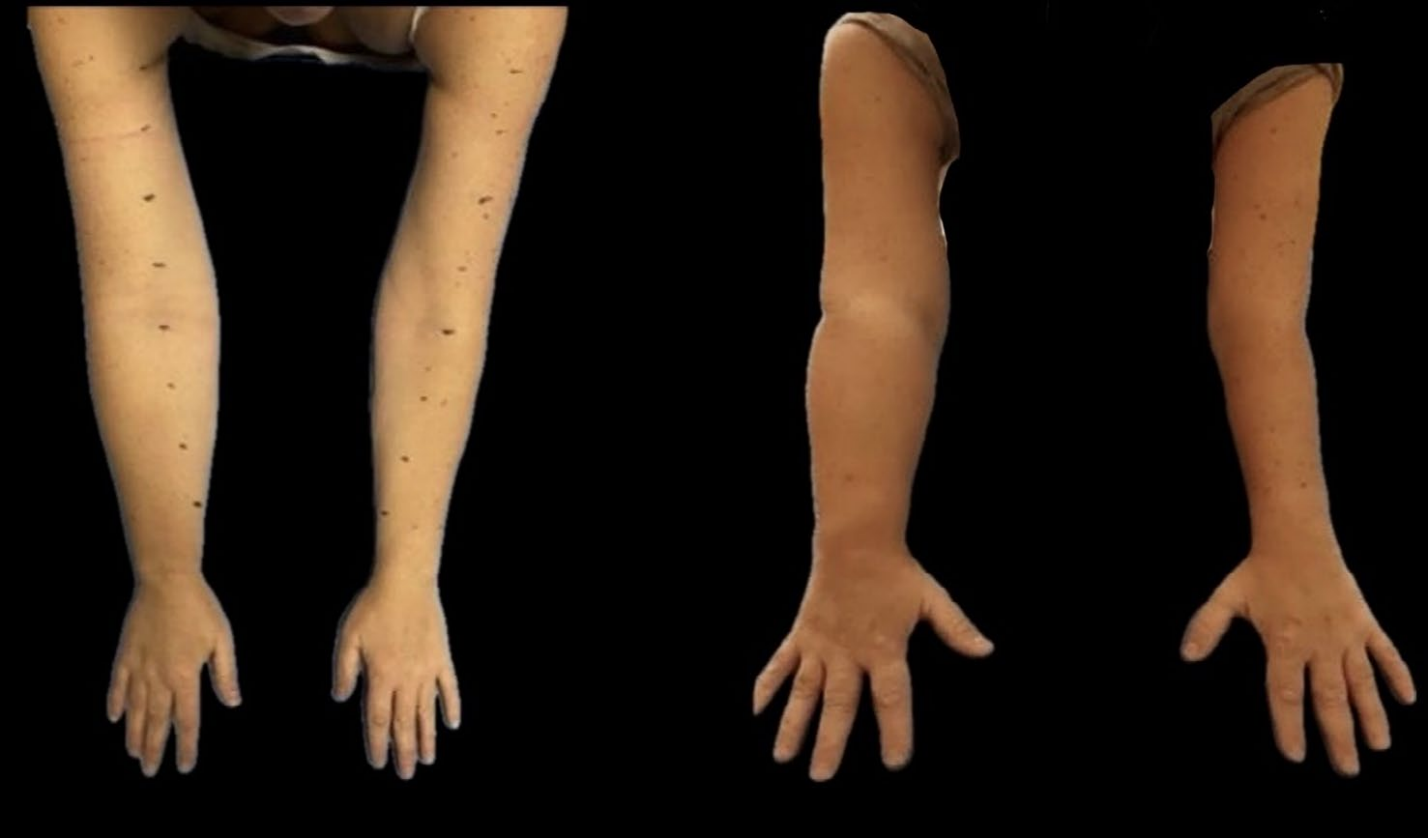
Preoperative (right) and postoperative (left) clinical photographs of a woman affected by breast cancer–related lymphedema (BCRL) of the left upper limb (ISL stage III) following mastectomy and axillary lymph node dissection. Lymphedema developed 12 months after oncologic treatment and was refractory to conservative management with complete decongestive therapy. The patient underwent vascularized lymph node transfer under conventional (non–ERAS‐VLNT) perioperative management.

**FIGURE 3:**
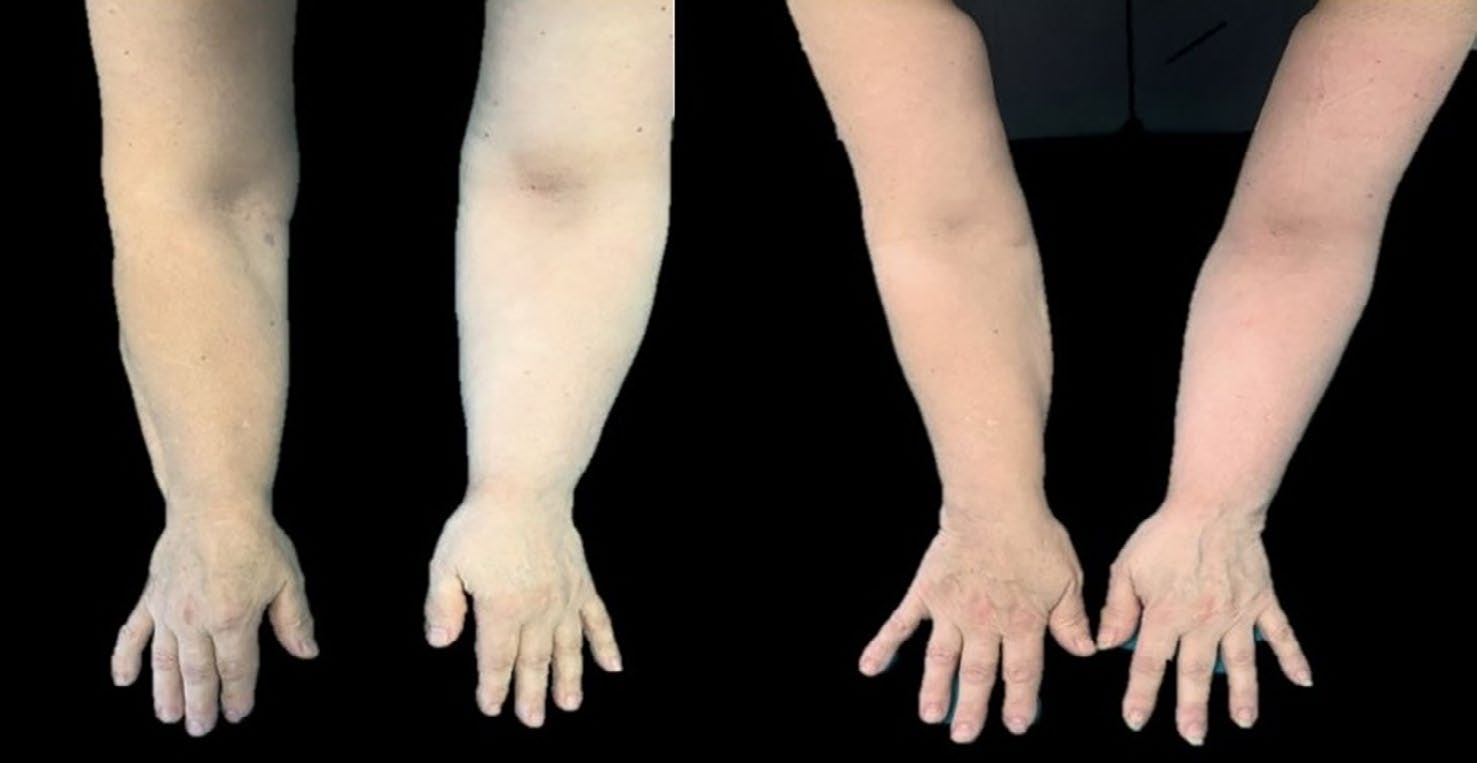
Preoperative (right) and postoperative (left) clinical photographs of a woman affected by breast cancer–related lymphedema (BCRL) of the right upper limb (ISL stage III) following mastectomy and axillary lymph node dissection. Lymphedema developed 9 months after oncologic treatment and was refractory to conservative management. The patient underwent vascularized lymph node transfer following the ERAS‐VLNT protocol.

The mean circumferential reduction rate (CRR) was 44.39% ± 10.20% in the ERAS group and 47.53% ± 8.26% in the control group (*p* = 0.278), showing no statistically significant difference between groups (Figures 2 and 3).

**FIGURE 4:**
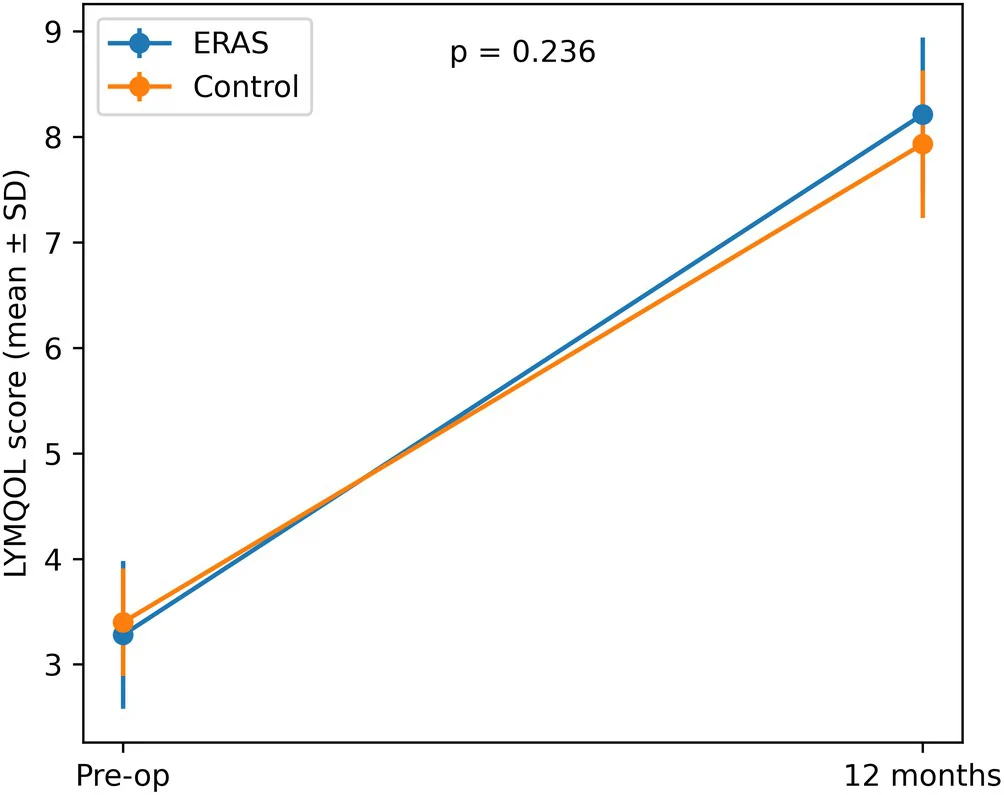
LYMQOL scores before and after VLNT. Comparison of LYMQOL scores between ERAS and conventional perioperative management groups at baseline and 12 months follow‐up. Values are expressed as mean ± standard deviation. Both groups demonstrated significant improvement over time, with no statistically significant differences between groups (*p* = 0.236), indicating comparable functional outcomes.

Similarly, patient‐reported quality of life assessed by LYMQOL improved substantially in both cohorts. LYMQOL scores increased from 3.28 ± 0.70 preoperatively to 8.21 ± 0.73 at 12 months in the ERAS group, and from 3.40 ± 0.51 to 7.93 ± 0.70 in the control group (*p* = 0.236) (Figure 4).

These results are in line with previously published VLNT series demonstrating significant improvements in both limb volume and quality of life following surgery (Lo Torto et al. 2024; Ciudad, Manrique, Bustos, Perez Coca, et al. 2020).

Soft tissue infection rates, expressed as episodes per year, were comparable between groups at 12 months (2.62 ± 0.90 in the ERAS group vs. 2.73 ± 0.96 in the control group, *p* = 0.71). Both groups showed a reduction in infectious episodes compared with preoperative values, reflecting the beneficial effects of VLNT on lymphatic function and immune response, as previously reported in the literature (Elia et al. 2024).

### 3.4 Perioperative Recovery Outcomes

Significant differences were observed in perioperative recovery parameters (Table 2).

**TABLE 2:** Clinical and perioperative outcomes.

Outcome	ERAS protocol (*n* = 29)	Control (*n* = 16)	*p*
Patient‐reported outcomes			
LYMQOL, preoperative	3.28 ± 0.70	3.40 ± 0.51	0.53
LYMQOL, 12 months	8.21 ± 0.73	7.93 ± 0.70	0.236
ΔLYMQOL (improvement)	+4.93	+4.53	—
Surgical outcomes			
CRR (%)	44.39 ± 10.20	47.53 ± 8.26	0.278
Flap survival, *n* (%)	29 (100%)	16 (100%)	—
Complications, *n* (%)	0 (0%)	0 (0%)	—
Perioperative recovery			
Length of stay (days)	2.34 ± 0.48	5.56 ± 0.51	< 0.001
Nasogastric tube, *n* (%)	0 (0%)	16 (100%)	< 0.001
Economic outcomes			
Estimated hospitalization cost per patient (€)	~1400	~3300	< 0.001
Estimated cost reduction per patient (€)	~1900	—	—

*Note:* Values are presented as mean ± standard deviation or number (percentage). CRR was calculated as the percentage reduction in limb circumference difference between the affected and contralateral healthy limb. Infection rates are reported as episodes per year.

Abbreviations: CRR, circumferential reduction rate; ERAS, Enhanced Recovery After Surgery; LYMQOL, Lymphedema Quality of Life questionnaire.

**FIGURE 5:**
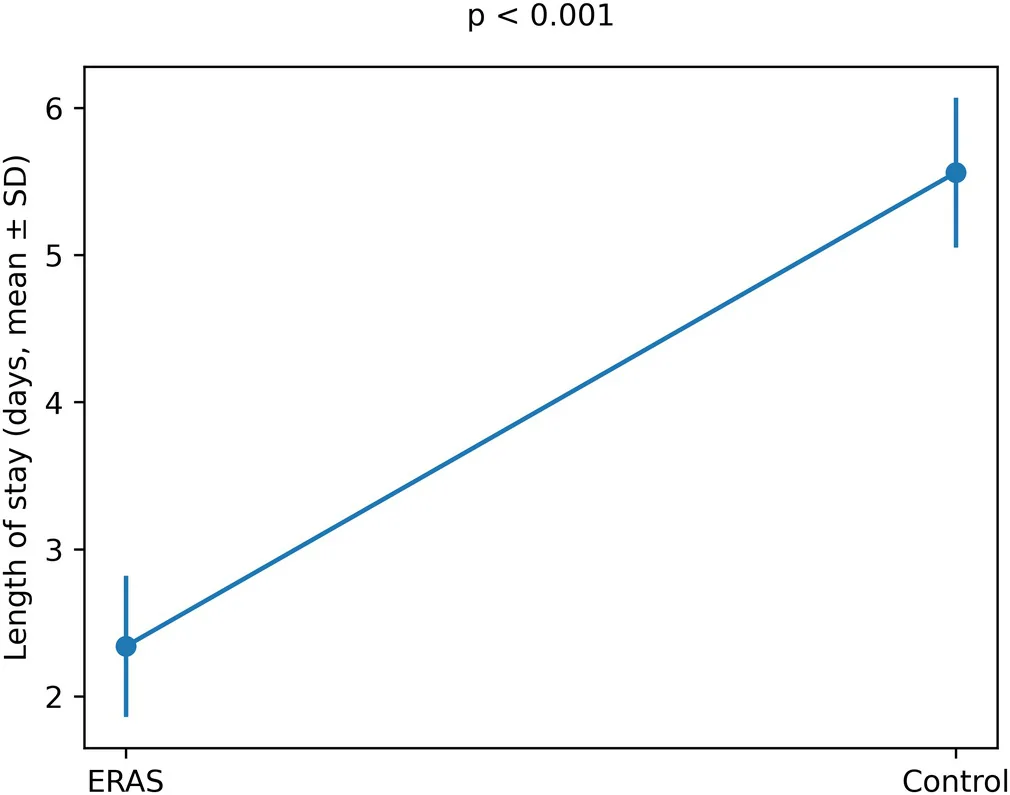
Length of hospital stay. Comparison of postoperative length of stay between ERAS and conventional management. Values are expressed as mean ± standard deviation. The ERAS group demonstrated a significantly reduced length of stay (*p* < 0.001), reflecting improved perioperative recovery.

The ERAS group demonstrated a markedly reduced length of hospital stay compared with the conventional group (2.34 ± 0.48 vs. 5.56 ± 0.51 days, *p* < 0.001) (Figure 5).

Nasogastric tube use was completely avoided in the ERAS group (0%) compared with 100% utilization in the control group (*p* < 0.001). In the conventional group, nasogastric tube removal was based on reduction of gastric output to less than 40 mL.

Early oral intake within 24 h was achieved in all patients in the ERAS group (100%), whereas it was delayed to approximately 48 h in the conventional group (*p* < 0.001).

Similarly, early mobilization from postoperative Day 1 was achieved in 100% of ERAS patients, whereas patients in the conventional group remained on bed rest for at least 48 h (*p* < 0.001).

Complete decongestive therapy (CDT) was initiated from postoperative Day 7 in the ERAS group, whereas it was delayed beyond postoperative Day 15 in the conventional group (*p* < 0.001).

No patients in the ERAS group required opioid administration, whereas opioids were routinely used in the conventional group.

### 3.5 Economic Outcomes

**FIGURE 6:**
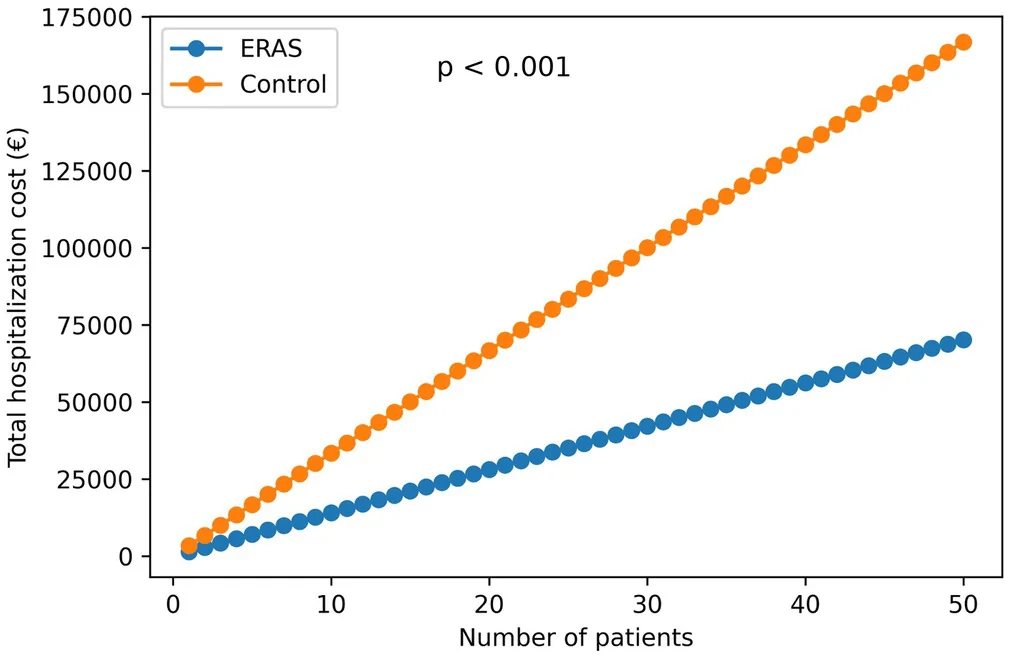
Hospitalization cost. Comparison of estimated hospitalization costs between ERAS and conventional perioperative management. The ERAS protocol was associated with a significant reduction in costs (*p* < 0.001), primarily driven by decreased length of hospital stay.

From an economic perspective, the reduction in hospital length of stay translated into a significant decrease in hospitalization costs. The estimated cost per patient was approximately €1400 in the ERAS group compared with €3300 in the control group (*p* < 0.001), resulting in an average cost reduction of approximately €1900 per patient (Figure 6). Cost estimates were based on average hospitalization costs derived from national healthcare data and should be interpreted as approximations, as indirect and societal costs were not included.

This reduction reflects the direct impact of ERAS implementation on healthcare resource utilization.

## 4 Discussion

The management of breast cancer–related lymphedema (BCRL) has undergone a profound evolution over the past two decades (Rockson 2018). Initially considered a chronic and largely irreversible condition, treatment was historically limited to conservative approaches such as complex decongestive therapy (CDT), often associated with suboptimal long‐term outcomes and significant impact on quality of life.

The introduction of physiologic microsurgical techniques, including lymphaticovenous anastomosis (LVA) and vascularized lymph node transfer (VLNT), marked a turning point in the management of lymphedema. VLNT, in particular, has emerged as a reliable option for advanced‐stage disease, demonstrating consistent improvements in limb volume reduction, infection rates, and patient‐reported outcomes (Cheng et al. 2013; Ozturk et al. 2016; Becker et al. 2006; Lo Torto et al. 2024; Ciudad, Manrique, Bustos, Perez Coca, et al. 2020).

Over time, several technical refinements have been introduced. Early VLNT procedures were predominantly performed using open techniques, particularly for gastroepiploic flap harvest, which were associated with increased donor‐site morbidity and prolonged postoperative recovery. More recently, the adoption of minimally invasive laparoscopic approaches has significantly reduced surgical trauma and improved postoperative recovery, as highlighted in recent series by Manrique et al. and Ciudad et al. (Manrique et al. 2020; Ciudad et al. 2017), representing an important advancement in microsurgical practice.

In parallel with these advancements, hybrid and combined surgical approaches have been proposed, including the integration of excisional procedures such as modified Charles techniques with physiologic strategies, as well as the combination of liposuction with lymphaticovenular anastomosis or vascularized lymph node transfer (Ciudad, Manrique, Bustos, Agko, et al. 2020; Ciudad et al. 2019). These approaches reflect an evolving trend toward personalized and multimodal management of lymphedema, particularly in patients with mixed fluid and adipose tissue components.

Similarly, the choice of inset site has evolved. While heterotopic (distal) inset gained popularity due to its technical simplicity and independence from proximal lymphatic pathways, orthotopic (axillary) VLNT has increasingly been recognized for its physiological advantages. By combining lymph node transfer with axillary scar release, orthotopic inset improves both venous and lymphatic outflow and may enhance functional outcomes (Lo Torto et al. 2024).

Despite these advances in surgical technique, perioperative management has remained largely unchanged and often overly conservative. In our previous institutional perioperative protocol, routine nasogastric tube placement, delayed oral intake, cautious mobilization, and relatively prolonged hospital stays were standard components of postoperative management following gastroepiploic vascularized lymph node transfer.

Previous series, including those by Ciudad et al. and other microsurgical groups, have reported mean hospital stays ranging from 4 to 7 days, reflecting a more conservative perioperative management often associated with open or non‐ERAS protocols (Manrique et al. 2020).

In parallel, the development of Enhanced Recovery After Surgery (ERAS) protocols has transformed perioperative care across multiple surgical specialties. Originally introduced in colorectal surgery, ERAS pathways have progressively expanded to include breast reconstruction and free flap microsurgery, where they have demonstrated reductions in complications, hospital length of stay, and healthcare costs, as shown in studies such as those by Bertelsen et al. (Bertelsen et al. 2020).

However, until now, these principles had not been specifically adapted to lymphatic microsurgery.

Unlike previously published ERAS pathways developed for reconstructive free flap surgery, the ERAS‐VLNT protocol incorporates several procedure‐specific elements, including laparoscopic gastroepiploic lymph node harvest, orthotopic axillary inset, flap monitoring adapted to buried lymph node flaps, early initiation of complete decongestive therapy, and integration of perioperative management with lymphatic rehabilitation. These features distinguish the present protocol from existing microsurgical ERAS pathways and represent its principal innovation.

Importantly, the implementation of the ERAS‐VLNT protocol did not compromise surgical safety. In our series, flap survival was 100%, with no cases of total or partial flap loss, no need for re‐exploration, and no donor‐site complications. These results are consistent with previously published VLNT outcomes, including those reported by Manrique et al. and Ciudad et al., confirming the reliability of gastroepiploic lymph node transfer with orthotopic inset (Manrique et al. 2020; Ciudad et al. 2017).

Functional outcomes, including CRR and LYMQOL scores, were comparable between ERAS and conventional groups, further supporting the safety of accelerated recovery pathways. These findings align with previous ERAS studies in microsurgery, where improved perioperative management did not negatively impact surgical outcomes (Bertelsen et al. 2020).

The most striking differences emerged in perioperative recovery. The ERAS‐VLNT protocol resulted in a dramatic reduction in hospital length of stay, decreasing from a mean of 5.56 days in the control group to 2.34 days in the ERAS group. This reduction is particularly relevant when compared with historical VLNT series, where hospital stays of 4–7 days have been consistently reported (Manrique et al. 2020).

Equally significant is the elimination of routine nasogastric tube use. While nasogastric decompression has traditionally been considered in abdominal procedures and was part of the conventional perioperative management in our earlier clinical practice, our results demonstrate that nasogastric tubes can be safely avoided without increasing gastrointestinal complications or requiring reinsertion. This represents a substantial shift in perioperative management.

Furthermore, early oral intake within 24 h and early mobilization starting from postoperative Day 1 were successfully implemented in all ERAS patients. These findings challenge long‐standing assumptions regarding the need for prolonged postoperative restriction and highlight the feasibility of applying ERAS principles even in complex microsurgical procedures.

Another key element is the early initiation of complete decongestive therapy (CDT), which was achieved starting from postoperative Day 7 in all ERAS patients. Traditionally, rehabilitation following VLNT has been delayed because of concerns regarding flap integrity. In our protocol, compression was applied to the affected limb while avoiding direct compression of the axillary region containing the transferred lymph node flap and microvascular anastomoses. This approach allowed early resumption of CDT without exposing the flap to mechanical stress. Early rehabilitation may also facilitate postoperative recovery by preventing prolonged interruption of lymphedema management. Importantly, no flap‐related complications were observed, supporting the safety of this strategy in the present series.

From an economic perspective, the implications of these findings are substantial. The reduction in hospital length of stay translated into a significant decrease in healthcare costs, with an estimated saving of approximately €1900 per patient. Cost estimates were derived from national healthcare data sources, including reports from the Italian Ministry of Health, the Ministry of Economy and Finance, and AGENAS (Ministero della Salute, n.d.; Ministero dell'Economia e delle Finanze – Ragioneria Generale dello Stato, n.d.; Agenzia Nazionale per i Servizi Sanitari Regionali (AGENAS), n.d.). Considering the increasing incidence of breast cancer‐related lymphedema and the growing demand for microsurgical treatment, the implementation of ERAS‐VLNT could translate into substantial cumulative savings at a population level. In addition, in the context of a chronic condition such as BCRL, which is associated with recurrent infections and long‐term healthcare utilization, this reduction represents a meaningful advantage for healthcare systems (DiSipio et al. 2013; Rockson 2018).

Overall, ERAS‐VLNT preserves surgical efficacy while improving perioperative efficiency and reducing healthcare resource utilization. These findings suggest that ERAS‐VLNT may represent an important step toward optimizing perioperative care in lymphatic microsurgery.

The main limitations of this study include its retrospective design, the relatively small sample size, and the chronological allocation of patients. The ERAS‐VLNT protocol was introduced in January 2023, resulting in a temporal separation between the study groups. Consequently, patients were allocated according to the period in which surgery was performed, introducing a potential temporal bias related not only to progressive perioperative optimization but also to increasing surgical experience over time. Although all procedures were performed by an experienced microsurgical team using a standardized technique, a learning curve effect cannot be completely excluded. Furthermore, the relatively small sample size may have limited the statistical power to detect significant differences in secondary clinical outcomes. Therefore, larger prospective multicenter studies are warranted to further validate the feasibility, safety, and effectiveness of the ERAS‐VLNT protocol.

However, the consistency of surgical outcomes, including a 100% flap survival rate in both groups, suggests that technical performance remained stable throughout the study period. Importantly, the adoption of the ERAS‐VLNT protocol was associated with a less invasive perioperative course, earlier hospital discharge, and reduced healthcare costs, without compromising surgical or functional outcomes. These aspects may be particularly relevant in a patient population already burdened by significant physical and psychological stress.

Further prospective and multicenter studies are warranted to confirm these findings and to define standardized guidelines for ERAS implementation in lymphatic surgery.

## 5 Conclusions

The implementation of an enhanced recovery after surgery (ERAS) protocol in gastroepiploic vascularized lymph node transfer is feasible and safe. The ERAS‐VLNT pathway allows a less invasive perioperative course, characterized by earlier mobilization, earlier oral intake, and avoidance of nasogastric tube placement, without compromising flap survival or clinical outcomes.

These findings support the integration of ERAS principles into lymphatic microsurgery and suggest potential benefits in terms of patient recovery and healthcare resource utilization. Given the limited sample size, these results should be interpreted as preliminary and warrant further validation in larger prospective studies.

## Funding

The authors have nothing to report.

## Conflicts of Interest

The authors declare no conflicts of interest.

## Supporting information


**Video S1:** Video representation of: laparoscopic harvest of the gastroepiploic lymph node flap; intraoperative view of the gastroepiploic lymph node flap with identification of the arterial and venous vascular pedicles; orthotopic inset of the previously harvested gastroepiploic lymph node flap into the axilla, with creation of end‐to‐end microvascular anastomoses; intraoperative assessment following completion of the end‐to‐end microvascular anastomoses using indocyanine green fluorescence imaging to confirm vascular patency.

## Data Availability

The data that support the findings of this study are available from the corresponding author upon reasonable request.
